# Oldest leaf mine trace fossil from East Asia provides insight into ancient nutritional flow in a plant–herbivore interaction

**DOI:** 10.1038/s41598-022-09262-1

**Published:** 2022-03-28

**Authors:** Yume Imada, Nozomu Oyama, Kenji Shinoda, Humio Takahashi, Hirokazu Yukawa

**Affiliations:** 1 Graduate School of Science and Engineering, Ehime University, 2-5 Bunkyo-Cho, Matsuyama, Ehime 790-8577 Japan; 2 Graduate School of Science, Kyushu University, 744 Motooka, Nishi-Ku, Fukuoka 819-0395 Japan; 3 Department of Construction, Agriculture, and Forestry, Mine City Office, 326-1 Higashibun, Omine-Cho, Mine, Yamaguchi 759-2292 Japan; 4 Mine City Museum of History and Folklore, 279-1 Higashibun, Omine-Cho, Mine, Yamaguchi 759-2212 Japan; 5 Fukui Prefectural Dinosaur Museum, 51-11 Terao, Muroko, Katsuyama, Fukui 911-8601 Japan

**Keywords:** Palaeontology, Evolutionary ecology

## Abstract

The Late Triassic saw a flourish of plant–arthropod interactions. By the Late Triassic, insects had developed all distinct strategies of herbivory, notably including some of the earliest occurrences of leaf-mining. Herein we describe exceptionally well-preserved leaf-mine trace fossils on a *Cladophlebis* Brongniart fern pinnule from the Momonoki Formation, Mine Group, Japan (Middle Carnian), representing the oldest unequivocal leaf-mines from East Asia. The mines all display a distinctive frass trail—a continuous meandering line, which later becomes a broad band containing spheroidal particles—demonstrating larval development. Although the shapes of the frass trails are generally comparable to those of Lepidoptera or Coleoptera, they cannot be unequivocally assigned to a specific extant leaf-mining taxon. Furthermore, elemental analyses by X-ray fluorescence (XRF) reveals that the frass trail comprises phosphate coprolites. The quantitative variations in P, S, and Si between coprolites and leaf veins may reflect physiological processes (e.g., consumption, absorption, and excretion) mediated by plant chemicals. Our findings reinforce the idea that leaf-mining had become a pervasive feeding strategy of herbivorous insects by the Late Triassic.

## Introduction

Leaf mining is a means of herbivory by which insects consume live foliage while dwelling inside the host-plant tissue^1^. The biology of leaf-mining has historically attracted much attention and is extensively studied by ecologists^2^. Extant leaf miners can be identified between species and subfamily level based on mine shape and host-plant taxonomy. Thus plant–insect associations can be reconstructed by analysing mined leaves. Mined leaves also provide information on much of the overall life history of an individual miner: namely, the developmental process, from oviposition, through larval growth and the pupal stage, to adult emergence, is externally traceable, which makes it an ideal system for the study of demography and population dynamics while modelling the rates and causes of mortality (e.g., parasitism, competition) in natural populations^3,4^. As leaf-mining insects afford a wealth of ecological information, they have frequently been used for studying population, community, and evolutionary ecology of plant–insect interactions^5–7^.

The evolution of leaf-mining is a compelling aspect of the development of dietary niches of insects. Insect groups with mining habits have evolved multiple times in several holometabolous insect orders (e.g., Lepidoptera, Coleoptera, Diptera, Hymenoptera)^1,8^. Miners are more susceptible to the chemical defences of host plants and are more severely attacked by parasitoids than external feeders^9^. Thus, they tend to show high host specificity, with many species being monophagous or oligophagous herbivores^2,10^, and they are embedded in a complex multitrophic network^11–13^. Some leaf-mining insect groups have been used for assessing patterns and processes of macroevolution within a phylogenetic framework^14–16^.

Leaf-mining has also been examined in palaeobiological studies because mine trails can be found from impression/compression fossils of plant leaves. In some cases, the leaf miner responsible for the mine is assignable to a specific insect group by comparing the shape of the fossil mine and the host-plant taxonomy with analogous extant ones^17–21^; these records can help to time-calibrate insect phylogeny. Furthermore, the specialized damage observed in fossil leaf mines enables us to answer various questions related to dynamic shifts or persistence of plant–insect interactions before and after ecological perturbations^22–25^.

Concerning the evolutionary origin of leaf-mining, however, available information is scarce. The earliest credible occurrence of leaf-mining so far dates back to the Middle–Late Triassic, which is much later than other functional feeding groups: namely, sporangivory, boring, external foliage feeding, seed predation, piercing-and-sucking, and galling^26,27^. Substantial evidence of leaf-mining is, however, very scarce in records preceding the Late Triassic.

Herein we describe a novel type of leaf-mine fossil from the Momonoki Formation, southwestern Japan. Described below are the exquisitely preserved mines in a *Cladophlebis* fern frond, and the traces preserve their organic remains. The specimen has already been displayed for years in a cabinet at the Mine City Museum of History and Folklore, Yamaguchi, Japan. The fossil locality is dated to be the Middle Carnian (ca. 220 Ma)^28^, and thus, the mines represent one of the oldest credible leaf-mine fossils worldwide. Additionally, elemental analyses using an X-ray fluorescence (XRF) spectrometer are conducted on the fossil mines, casting new light on this ancient plant–herbivore association from the perspective of ecological stoichiometry.

## Materials

### Geological setting

**Figure 1:**
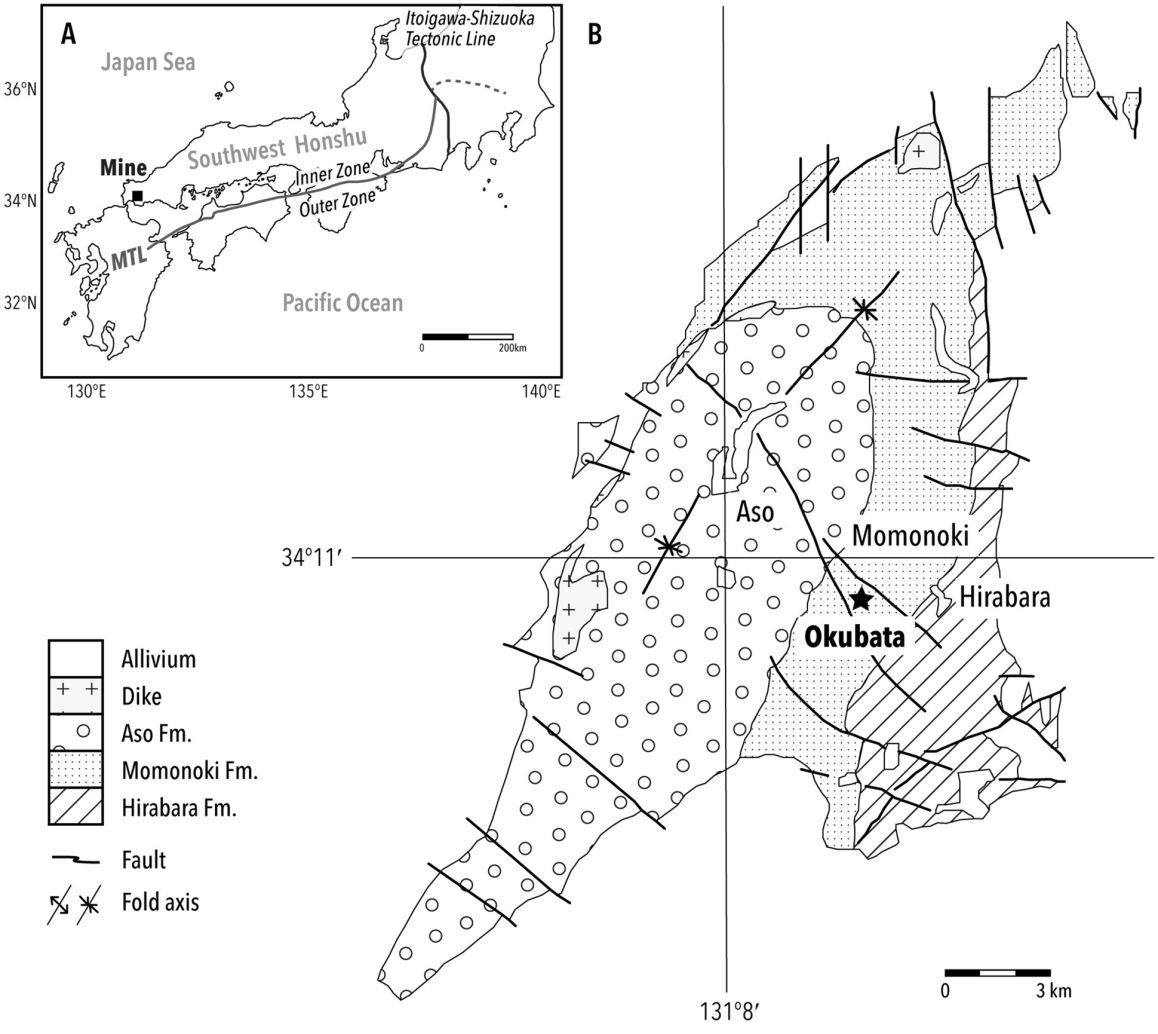
Study site location and geological map. (**A**) Map showing the fossil site, Mine, Yamaguchi, in southwest Honshu, Japan. Mine is located in the Inner Zone, the region formed by complex faulting along the Median Tectonic Line (MTL) and separated from the Outer Zone (southern side). (**B**) Geological map of the Mine Group, slightly modified from previous studies^38,39^. The star denotes the fossil locality, Okubata.

The Mine Group, which faces continental China, is located on the northern side of the Median Tectonic Line, which divides the southwest end of Honshu island, Japan (Fig. 1). The stratigraphic setting is subdivided into three strata: the Hirabara, Momonoki, and Aso Formations, in ascending order^29,30^. Study material was obtained from the Momonoki Formation, which yields the most abundant fossil plants and insects^31,32^. The Momonoki Formation is dated to the Middle Carnian (ca. 220 Ma) based on U–Pb age data of detrital zircon^28^, which is consistent with the biostratigraphy of marine and terrestrial invertebrates^29,33^. The Momonoki Formation exceeds 1000 m in thickness and is conformable with the subjacent Hirabara Formation^28,32^. The sandstones and muddy sandstones of the Momonoki Formation host impressions of plant and insect macrofossils. The specimen was collected by H.T. at a road cutting on National Route 435 while the road was under construction; the locality also bore many insect fossils^32,34–36^. The Momonoki Formation is a nonmarine deposit characterized by lacustrine, deltaic deposit without marine invertebrate fossils, unlike in the Hirabara and Aso Formations^31,37^.

### Palaeobiological setting

Plant remains from this locality are primarily represented by impressions of foliage, stem fragments with leaves, and, occasionally, fructifications. The plant assemblage of the Momonoki Formation is composed of ferns, sphenophytes, cycadophytes, ginkgophytes, and conifers^40^. There are three characteristics of the Momonoki Formation that are distinct from some other coeval fossil plant assemblages^41^: Sphenopsida (e.g., *Neocalamites* Halle and *Equisetites* Sternberg), ferns (e.g., *Camptopteris* Presl, *Clathropteris* Brongniart, *Dictyophyllum* Lindley et Hutton, *Cladophlebis*), and conifers (e.g., *Podozamites* Braun, *Cycadocarpidium* Nathorst) are well represented; cycadophytes and ginkgophytes^42^ are common; *Danaeopsis* Schimper and *Symopteris* Xu (Marattiaceae) ferns are lacking. Liverworts (*Pallaviciniites* Schuster) are also present^43^. The floral composition of the Momonoki Formation is part of the Southern Floristic Region of East Asia^41,44^; the floristic region extends over a broad geographic range, from as far south as Indonesia and north to a small area of northeastern China, and is characterized by the occurrence of *Dictyophyllum* and *Clathropteris*^41^.

A diverse fossil insect fauna has been found at the Momonoki Formation^36,45^. These fossils are borne from specific stratigraphic layers of dark, fine-grained siltstones that are devoid of coarse detritus^31^. Most of the insect fossils are isolated wings, but nearly complete bodies are also found^36^. The recorded insects encompass at least 12 orders: Odonata, Ephemeroptera, Blattodea, Plecoptera, Paraplecoptera, Reculida, Neuroptera, Hymenoptera, Coleoptera, Hemiptera, Mecoptera, Diptera^32^. In particular, there are rich yields of wings of Coleoptera (Taldycupedidae) and Blattodea (Mancusoblattidae, Mesoblattinidae) amongst the insect fauna of this deposit^32,34^. Notably, the Momonoki Formation yields true flies^36^, albeit undescribed, which have only been recorded from a few deposits from the Middle–Late Triassic^46–51^. Although more than 6000 specimens of fossil insects are recorded from this formation^30,36^, the taxonomic diversity is largely unexplored, and numerous terrestrial and aquatic insect specimens remain undescribed. At present, 18 species in ten families belonging to seven orders are described^32–35,45^. The insect fauna of the Mine Group is thought to have an affinity with four remote localities^32,33^: the Mount Crosby Formation in Australia, the Madygen Formation in Kyrgyzstan, the Djam Djun Formation in Vietnam, and the Yan-Chen Formation in China.

## Results

### Description of trace fossil

**Figure 2:**
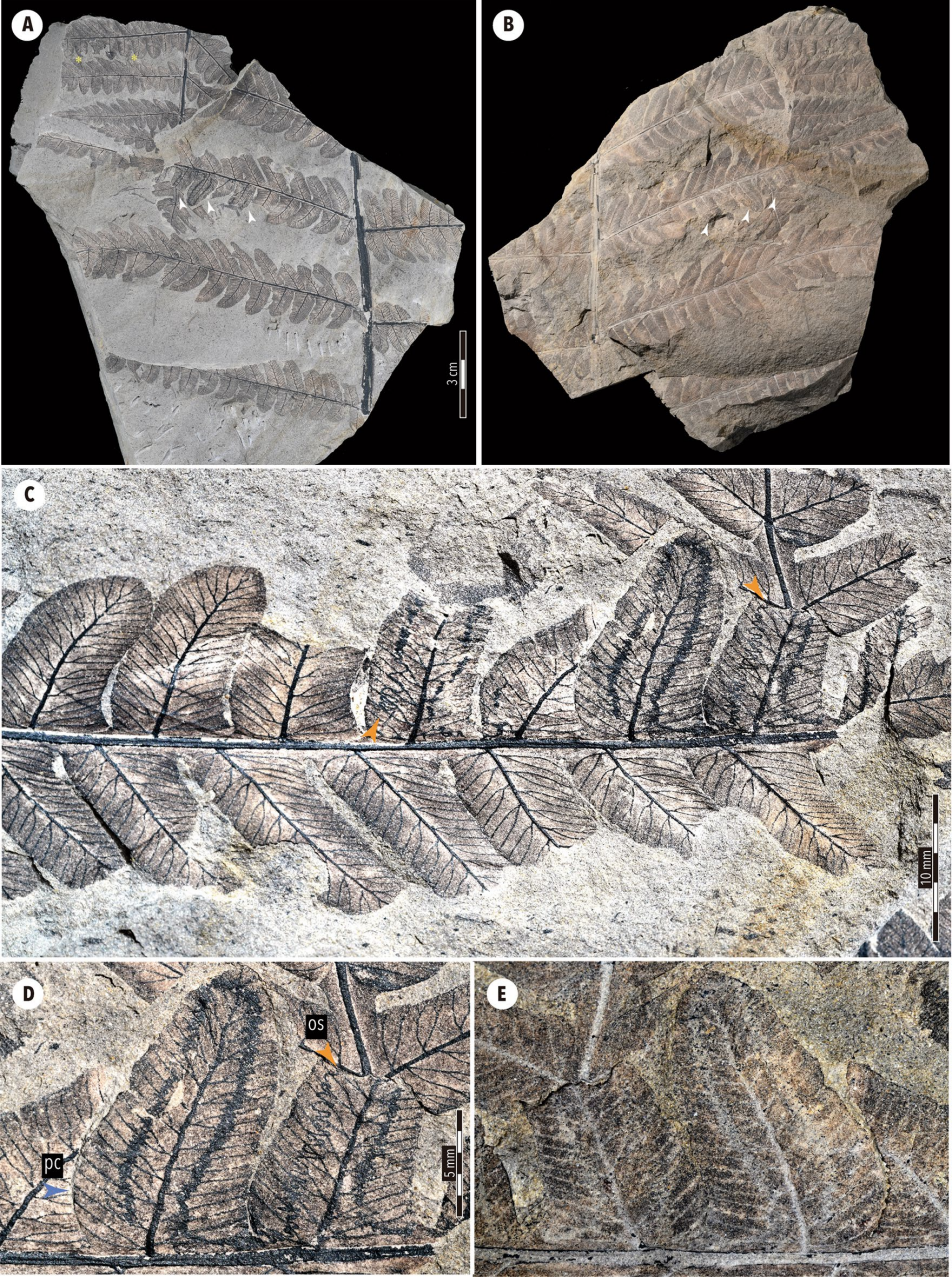
Late Triassic leaf-mine fossil on *Cladophlebis denticulata* of the Momonoki Formation, Yamaguchi, Japan. (**A**) Specimen (MMHF11-00001a), on which mines on pinnules and chewing marks are marked with white arrowheads and yellow asterisks, respectively, and (**B**) its counterpart (MMHF11-00001b), at approximately the same scale. (**C**) Enlargement of pinna with three mines; orange arrowheads signify putative starting point (i.e., oviposition site) of leaf-mines; however, the starting point of the mine on the right is unseen. (**D**) Enlargement of two pinnules shows the transition of the frass trail, suggesting larval development while mining, and (**E**) its counterpart at the same scale. Arrowheads in orange and blue denote an oviposition site (os) and possible pupal chamber (pc), respectively.

Three leaf mines with similar structures are found on five consecutive pinnules of the basiscopic pinna (white arrowheads in Fig. 2A,B). The plant tissues, including the epidermis and veins, stand out sharply against the profoundly weathered matrix of the slab. The mines broadly cover the lamina, although the margins of mined and remaining areas are not clearly distinguished as breached epidermal tissue, indicating that the miners left some tissue layers unconsumed. The mines originate either from the proximal or distal end of the lobes. Starting points (i.e., oviposition site) are obscure or unseen (orange arrowheads in Fig. 2C,D), lying on a secondary leaf vein. Frass trails appear as narrow sinusoidal threads with a width of 0.5–1.3 mm (1.1 mm on average), accompanying many close-set hairpin curves with occasional loops. The frass trails subsequently expand into broad bands of width 0.5–0.8 mm, with the dense accumulation of faecal material; individual frass pellets are hardly discernible. The frass trails are single, continuous lines but accompanied with a few gaps; altogether, each frass trail covers nearly the entire distal part of a pinnula. Although the trajectories of the frass trails freely extend across the secondary veins, they do not traverse the first veins (i.e., midribs) except those near the leaf apex; such behavioural characteristics thus give the entire mining structure on a pinnule a U-shape. These mines connect to the neighbouring pinnule through the widened base of the pinnule (Fig. 2D–E). The fact that the leaf veins within the mined area are not distorted dictates that the vascular bundles are left unconsumed. The darkened tissue at the end of the mine indicates a possible pupal chamber (pc, Fig. 2D).

### Locality

Okubata, Omine, Mine, Yamaguchi.

### Age

Carnian, Triassic (ca. 220 Ma).

### Stratigraphy

Momonoki Formation, Mine Group, Yamaguchi prefecture, Japan.

### Material

A single part-and-counterpart specimen (MMHF11-00001a, MMHF11-00001b; Fig. 2A,B, respectively) collected by H.T. from National Route 435 while the road was under construction (Fig. 1).

### Host plant

The host plant is considered to be *Cladophlebis nebbensis* (Brongniart) Nathorst based on the following characteristics. Rachis 3 mm wide, grooved adaxially and rounded abaxially. Pinna subopposite, attached by a broad base to the rachis at an angle of 70–75 degrees, with internodes of 34 mm; apex unknown. Leaves sterile and bipinnate. Pinnules sessile, subopposite, arising at an angle of 71–81 degrees to the pinna rachis, catadromous in order; acroscopic pinnules slightly longer and narrower than basiscopic ones; close-set, occasionally overlapping. Pinnule about 11–14 mm long and 7–8 mm wide (surface area ca. 91.9 mm^2^), with widened base and obtuse apices; veins catadromous, having distinct primary vein reaching apical margin and 11–13 secondary veins forking once. This species may alternatively be *Todites fukutomii* Kimura et Ohana^52^, but this possibility is not considered here because some distinguishing characters (e.g., fertile pinnules and twice-forked secondary veins) are lacking in our specimen.

### Remarks

The focal *Cladophlebis* frond is targeted by external foliage feeders in addition to miners. Excisions bordered by evident reaction rims (the area defined by two yellow asterisks in Fig. 2A) indicate signs of external feeding by a mandibulate folivore.

### X-ray fluorescence (XRF) analyses

**Figure 3:**
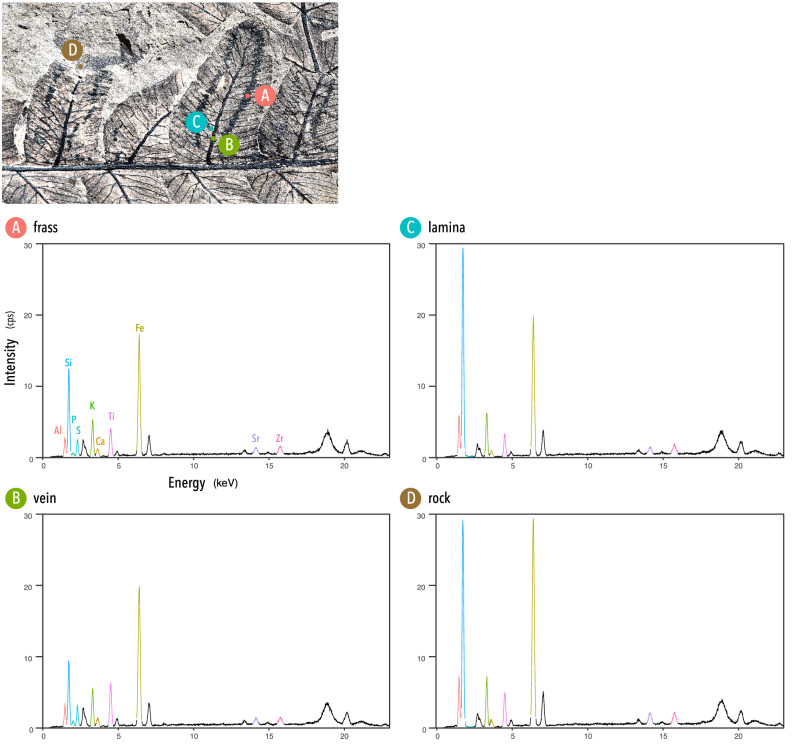
Results of X-ray fluorescence (XRF) analyses conducted with an EA6000VX High Sensitivity XRF Analyzer (Hitachi High-Tech Science Corporation). (Upper panel) Positions of the four sample points are specified on the specimen. (Lower panels) XRF spectra in the range of 0–23 keV illustrated for each sample point (**A**–**D**). Vertical axes signify cps (X-ray counts per sec).

Elements were quantified at four sample points: (A) frass trail, (B) leaf vein, (C) leaf lamina, (D) rock matrix (Fig. 3). The XRF spectrum detected peaks of ten elements (Al, Si, P, S, K, Ca, Ti, Fe, Sr, Zr); the other peaks were of Rh gas used for the analyses. The values for four elements (Si, P, S, Ca) were particularly notable because these elements could be at least partly biogenic and involved in physiological processes. The leaf lamina was composed of just a thin film of plant tissue, and thus the elemental composition might include that of rock beneath the lamina. The results for the leaf lamina generally showed a similar trend with that of rock; however, P and S, presumably of biogenic origin, were detected, confirming that leaf tissue was preserved on the rock. We report the results below as the total amount of X-ray per sec (cps). Marked variations were found in Si; it was highest in the leaf lamina (376.457 ± 5.394 cps), slightly higher than in rock (366.144 ± 5.323 cps), and lowest in the leaf vein (114.689 ± 3.029 cps). The highest P was recorded in the leaf vein (11.680 ± 1.149 cps), which was followed by P in frass (7.923 ± 0.992 cps), whereas peaks for P were not detected in the lamina or rock. S showed a similar trend as P, but with peaks in frass (29.034 ± 1.614 cps) and the vein (11.680 ± 1.149 cps) being higher than those of P. Ca was consistently detected from all measured sample points and showed only minor differences among them; it was highest in the leaf vein (19.380 ± 1.381 cps), followed by frass (17.519 ± 1.286 cps), rock (16.851 ± 1.251 cps), and leaf lamina (13.895 ± 1.158 cps).

## Discussion

### Significant features of the mines and the possible culprit

The *Cladophlebis* mining structures from the Momonoki Formation are leaf mines by holometabolous insects and can be distinguished from other feeding methods (e.g., surface-feeding) and also from features of taphonomic origin because they more or less satisfy the following criteria for shapes of insect leaf-mines^53^: an oviposition site at one end of the mining structure; an enlarged oviposition area as a blotch or curvilinear trace; a sign of the evacuated leaf tissue; width of evacuated plant tissue and frass trail increases from one end to the other; the presence of a frass trail, either particulate or fluidized; response tissue along with the marginal tissue; a distinctive terminus, such as an expanded region (chamber). In this case, features of the frass trails are shared by the three mines on the same pinna (Fig. 2A,B), which are most likely to be produced by the same insect taxon who has a stereotyped strategies for consumption and excretion. Each mine is composed of a continuous, single frass trail, the width of which subsequently expands at the end. The abrupt change in the shapes of faecal tracks can be interpreted as larval development while mining. In the mined pinnules, boundaries between mined and unmined areas are obscure because the frass trails frequently cut across the secondary leaf veins without distorting them. This can be interpreted in two ways: the larvae might mine only epidermal cells and they did not consume mesophylls, as in the case of *Phyllocnistis* Zeller (Lepidoptera: Gracillariidae)^54,55^; otherwise, the larvae could mine mesophyll avoiding vascular tissues (e.g., some agromyzid flies^56^). The latter strategy is possible because the mesophyll of ferns is anatomically well-differentiated into palisade and spongy tissues^57^.

Mine morphology and host-plant range often provide us with keys to identify leaf-mining insects because leaf-mining insects tend to be associated with a relatively narrow range of plants and show stereotyped, taxon-characteristic behavioural patterns in oviposition and feeding^2^. However, in general, the convergence among different insect orders/families and variation among closely related species make it difficult to differentiate miners based on their mine shapes^53^.

The taxonomic affinity of the mines from the Momonoki Formation is herein examined in the light of mine shape, systematic and evolutionary backgrounds of leaf-mining clades, the extant groups of fern-pinnule miners, and the chronological origins of possible culprits. The overall mine shape is not comparable to those of known mining structures by extant fern-miners^58^. Extant fern-mining insects are found in four orders: Diptera, Coleoptera, Lepidoptera, and Hymenoptera^59^.

Leaf-mining flies are diverse, encompassing three infraorders, Culicomorpha (e.g., Chironomidae), Bibinomorpha (e.g., Sciaridae), and Muscomorpha (Empidoidea–Muscoidea). Among them, Agromyzidae contain an overwhelming number of leaf-mining taxa. No evidence for the presence of dipteran leaf-mining taxa is available for the Late Triassic, although nematocerans and some of the earliest groups of brachycerans are markedly diverse^46,47^. Typical leaf mines of Agromyzidae, to which all fossil dipteran leaf mines have been assigned^60^, make linear-blotch mines^61^, and mine through mesophylls leaving major veins^62^; they tend to contain fluidized frass, which is often deposited as two discontinuous rows of pellets^19^. However, the forms of leaf mines by Agromyzidae greatly vary among taxa^63^. Flies are relatively diverse as miners of fern pinnules (and stems), represented by *Chirosia* Rondani (Anthomyiidae)^64^, Agromyzidae (e.g., *Chromatomyia* Hardy, *Phytoliriomyza* Hendel), and Cecidomyiidae^59^. Among them, *Chirosia* is a predominant component that use fronds or stems of a range of fern taxa^58,65–70^. In mines of some species of *Phytoliriomyza,* the frass trail displays a shift from a narrow meandering line to a wider band of the faecal pellets, which, to some extent, resemble those of the mines from the Momonoki Formation.

Leaf-mining taxa of Hymenoptera are mainly composed of sawflies (Tenthredinidae). This order was already diverse by the end of the Triassic^71^, with the oldest fossils dating back to the Middle Triassic^72^. Leaf-mining sawflies produce large conspicuous blotch mines^53,73^ and the larvae deposit cylindrical frass pellets which are scattered irregularly about the mine cavities^58,74^. The extant fern-feeders of Hymenoptera do not include pinnule-miners, and only Blasticotomidae and Tenthredinidae, as petiole-borers and internal fern-feeders, respectively, are known^59^.

The coleopteran leaf miners are known from Buprestoidea, Chysomeloidea, Curculionoidea, and a few other clades^60^; fern-pinnule mining taxa are reported from these superfamilies^59^. Beetles became widespread worldwide in the Middle to Late Triassic^75–77^. The earliest beetle group, Protocoleoptera, are found from the Momonoki Formation^34,45^, although they are thought to be saproxylic (i.e., borers of decaying wood), based on some circumstantial evidence^78^. Linear mines containing granular faecal pellets from the Triassic are often assigned to beetles, e.g., Polyphaga^79^. Notably, buprestid mines have some distinctive features that are comparable to those of the described mines. The buprestid mines are typically blotchy; they often are full-depth mines, avoiding epidermal and vascular tissues; the frass trails are generally long and stringy when freshly deposited, which later can be fragmented and become granular^58,80–83^.

Lepidopteran leaf-miners mainly consist of microlepidopteran groups, including Nepticuloidea, Gracillaroidea, and part of Yponomeutoidea. Based on a recent fossil-calibrated molecular phylogeny^84^, the appearance of leaf-mining moth clades, represented by the split between Nepticulidae and Opostegidae, dates back to the Late Jurassic; for calibrating this phylogeny, wing-scale fossils of Coelolepida from the Triassic–Jurassic boundary of Germany were used^85^. The earliest presumed nepticulid leaf mine fossils are known from the Dakota Formation of the Early Cretaceous (102 Ma)^18,86,87^. The lepidopteran mines exhibit considerable variation in mine shape, tissue consumption, and contents (e.g., faecal pellets). Notably, leaf mines of *Ectoedemia* (Nepticulidae) typically start as fine, strongly meandering galleries that subsequently become broad blotches^87^. In addition, typical nepticulids generally leave granular pellets, with abrupt changes in the accumulation pattern in some species. Importantly, these mine features are seen in the *Cladophlebis* mine from the Momonoki Formation. The Gracillariid moths produce serpentine or blotch mines and the mode of leaf-mining is conserved at the subfamily level^88^. Many gracillariid taxa (Acrocercopinae, some Gracillariinae, and Ornixolinae, and Lithocollectinae) make a narrow linear mine during the sap-feeding phase of early instars which later become a simple blotch mine. With regard to the fossil and molecular evidence, it is therefore unlikely that a member Nepticulidae caused the Late Triassic mine, although the Nepticuloidea or another early leaf-mining moth group cannot be ruled out.

Altogether, the shape of the *Cladophlebis* mines does not conform to typical mines of Diptera (Agromyzidae) and Hymenoptera; instead, it shares more features with those of Coleoptera or Lepidoptera in terms of the mine shapes. The assignment of a fossil leaf mine to a particular taxonomic group of insects is subject to uncertainty and limitations^19^. Available biological accounts of leaf-mining insects are limited, and leaf mines are often not photographed or illustrated. Particularly, in this case, a series of diagnostic features—oviposition habit (e.g., oviposition scar, deposition of eggs), pupal chamber, mining tissue types (e.g., epidermis, parenchyma), relative position of the frass trail in the mine—were not readily recognizable. Furthermore, the mine recorded herein predates the estimated divergence time of major clades of leaf-mining insects in the modern, as examined above. Overall, the potential leaf-miner may be Coleoptera or Lepidoptera, in terms of the general resemblance of the mine shape, the time of appearance, and the presence of records at the order level, although the possibility of Hymenoptera and Diptera is not completely excluded, due to the lack of conclusive diagnostic features. Future taxonomic studies on the insect fossils from the same deposit would provide support for ascertaining the suspect leaf-miner.

### The stoichiometric footprint of the studied plant–insect interaction

The elemental analyses indicate quantitative variability in some elements (Si, P, S) that may partly be responsible for physiological processes in nutritional cycles (Fig. 3); plant tissues are deposited and then partly removed by an insect and then the insect metabolizes and excretes the undigested substances. The content of frass is thus the product of absorption, metabolism, and excretion. Compared to the leaf vein, the fossilized frass (coprolites) are shown to be highly phosphatic, which is consistent with the fact that the coprolites are largely composed of calcium phosphate.

Another notable point is the varying intensity of Si among sample points. For the leaf lamina, biogenic and lithologic Si may be conjugated, and thus caution is needed in interpreting our result that the highest intensity of Si was found in the leaf lamina. However, the incremental difference of Si between the frass and leaf vein may be the result of biogenic silica contained in the frass. Biomineralization of silica, especially in the form of phytoliths (SiO_2_, nH_2_O), is found in many clades of pteridophytes^89,90^, and these phytoliths can enhance plant resistance to herbivore feeding^91,92^. This relatively higher content of Si in the frass coprolites may therefore reflect undigested defensive compounds.

This study illustrates that ecological stoichiometry, a method that traces the flow of energy and elements in ecosystems, can be applied to interactions between plants and endophytic herbivores which occurred 220 million years ago. Similarly, the synchrotron X-ray Fluorescence (SRS-XRF) analyses for exceptionally preserved leaf fossils of *Acer pseudoplatanus* L. from the Green River Formation (Eocene, ca. 50 Ma)^93^ have revealed a high concentration of some metal elements (e.g., Cu, S, Zn) in the plants and the faecal materials; however, phosphorus in the leaves was below the detection limits. Future investigations of plant chemical landscapes (nutrition and defence) across various temporal and spatial settings would provide new insights into the macroevolutionary patterns of combat between plants and herbivores.

### Origin and early history of leaf-mining

Credible leaf mines are absent or very rare before the Late Triassic^27^. From the Palaeozoic, at least two types of trace fossils have been previously assigned as possible leaf mines, although this is currently not supported. One type concerns U- or V-shaped structures on pinnules of medullosans from the Upper Carboniferous, or Lower Permian^94,95^, which were later attributed to fungal or bacterial infection^27,96,97^. The other types represent a series of small and extensive serpentine structures^94,98,99^, the ichnotaxon names of which are *Asteronomus meandriformis* Müller and *A. divergens* Müller; currently, these are acknowledged as structures of taphonomic origin^27^. A notable example from the Early Permian is a possible mine on a megaphyll of *Glossopteris* cf. *indica* from the Rio Bonito Formation, Morro do Papaléo Mine, Brazil; this structure contained the possible frass of the miner and ended with a terminal expansion, which was possibly a larval/pupal chamber^100^. Another example is a U-shaped contour on the foliage of *Vjaznikopteris rigida* Naugolnykh from the Volga River Basin (the P–Tr boundary), European Russia^101^. However, the identity of these traces as leaf mines is disputable because the above-mentioned criteria for insect mines are not met.

**Figure 4:**
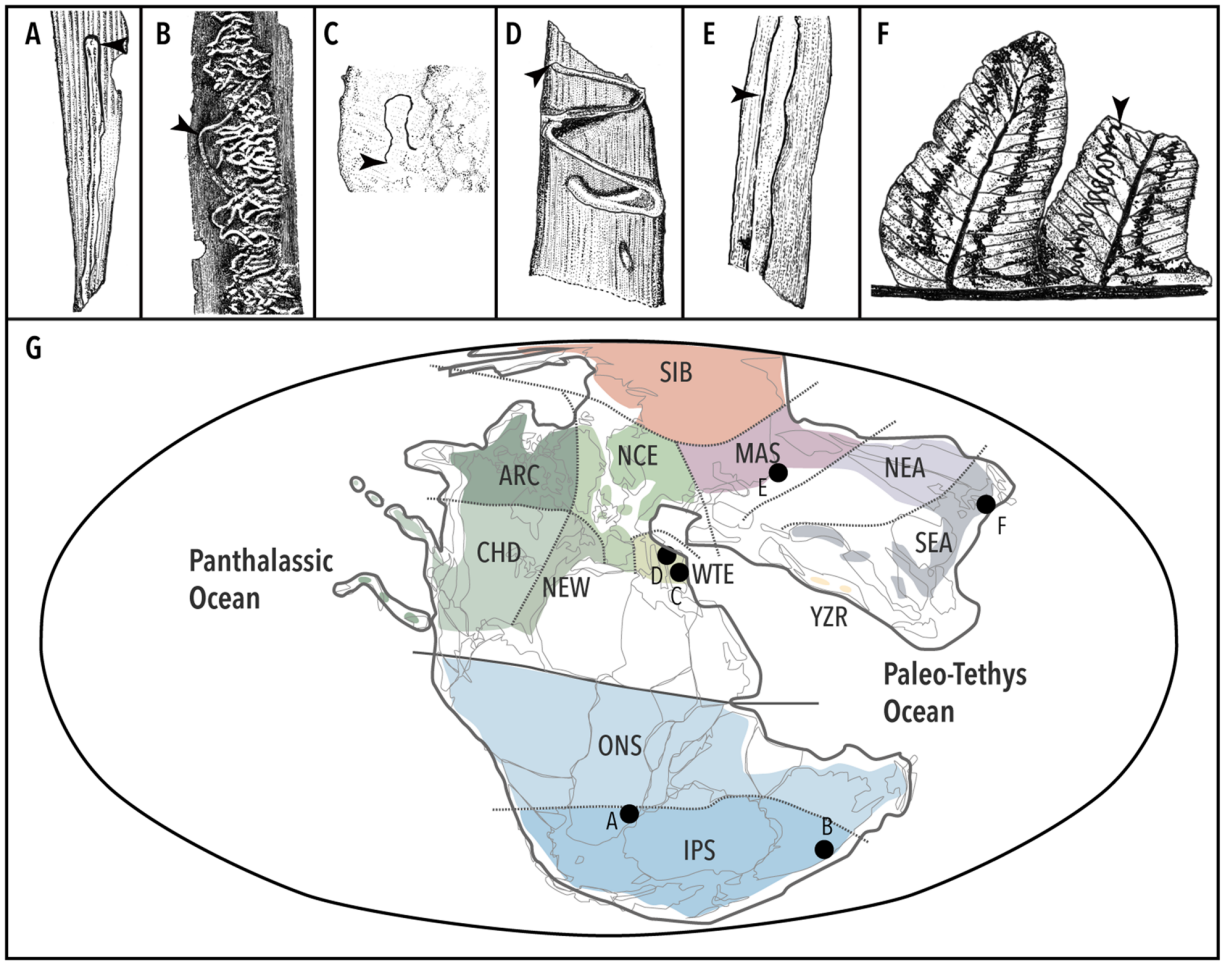
Mining structures known so far from the Middle–Late Triassic. (**A**) *Heidiphyllum* foliage mine (DT71) from the Molteno Formation (Carnian)^79^; two other recorded mines (DT41, DT139) from this formation are unillustrated. (**B**) *Heidiphyllum* foliage mine, *Triassohyponomus dinmorensis*, from the Blackstone Formation (Carnian) of the Ipswich Coal Measures Group, Queensland, Australia^105^. (**C**) Pteridosperm foliage mine from Monte Agnello, N. Italy (Late Ladinian)^106^. (**D**) *Nilssonia* foliage mine from the Lunz Formation (Carnian), eastern part of the Northern Calcareous Alps, Austria^107^. (**E**) Gymnosperm foliage mine from Dzhayloucho (Ladinian–Carnian), near Madygen, Kyrgyzstan^108^. (**F**) *Cladophlebis* pinnule mines from the Momonoki Formation (our study). (**G**) Paleogeographic map of the Late Triassic (Carnian) and the approximate locations of fossil mine localities are shown; floral zonation is based on a previous study^44^. Colours and three-letter acronyms represent floral zones as follows: *SIB* Siberian Subprovince, *MAS* Middle Asian Subprovince, *NEA* Northern East Asian Subprovince, *SEA* Southern East Asian Subprovince, *ARC* Arctic Canada Subprovince, *NCE* North Atlantic/Central European Subprovince, *NEW* Newark Subprovince, *CHD* Chinle/Dockum Subprovince, *WTE* Western Tethydean Subprovince, *YZR* Yarlung-Zangbo-River Subprovince, *ONS* Onslow Subprovince, *IPS* Ipswich Subprovince. Line drawings were made by Y.I. with Adobe Illustrator® 2021.

From the Middle–Late Triassic, several distinct shapes of mining structures are known (Fig. 4), and some of them are described as distinct damage types (DT)^102^. Several sites of the Molteno Formation (Carnian) are a rich source of herbivory and oviposition trace fossils^79,103^. Two types of leaf mines, one of each from *Heidiphyllum elongatum* (Morris) foliage (DT41, DT71) and one from *Sphenobaiera schenckii* (Feistmantel) Florin (DT139), have been recorded from this locality^79^ (Fig. 4A); additionally, this locality bears an undescribed, well-preserved mine on *Cladophlebis* fern pinnules (Labandeira, C. C., *pers. comm.*), although its shape differs considerably from the one from the Momonoki Formation. Another record from the Gondwanan flora is a serpentine mine on *Heidiphyllum* Retallack foliage, *Triassohyponomus dinmorensis* Rozefelds et Sobbe*,* from the Blackstone Formation (Carnian) of the Ipswich Coal Measures Group, Queensland, Australia^104,105^ (Fig. 4B). Some trace fossils from several other localities of the Middle–Late Triassic have also been assigned to leaf mines; for example, a small, semilinear, frass-laden mining structure (DT40) on foliage of the pteridosperm *Scytophyllum bergeri* Bornemann, from Monte Agnello, N. Italy (Late Ladinian)^106^ (Fig. 4C). Some mining structures are also known from the foliage of *Nilssonia sturii* Krasser from the Lunz Formation (Carnian), in the eastern part of the Northern Calcareous Alps, Austria^107^ (Fig. 4D); also, two types of mines on some gymnosperm (?*Glossophyllum*) foliage are reported from Dzhayloucho (Ladinian–Carnian), near Madygen, Kyrghyzstan^108,109^ (Fig. 4E).

The *Cladophlebis* mines (Fig. 4F) described here represent a novel damage type that serves as the oldest credible fossil mine from the Southern Floristic Region of East Asia, the palaeobotanical assemblage of which is geographically and taxonomically distant from any of the above-mentioned floras (Fig. 4G). Our finding, therefore, reinforces the view that leaf-mining had become a pervasive feeding method for plant-feeding insects by the Late Triassic. By this time, they had already colonized a wide range of plant groups: conifers, pteridosperms, cycadophytes, ginkgophytes, and ferns.

## Methods

Plant–arthropod interactions were censused for ca. 200 full storage boxes (ca. 536 × 336 cm) of fossil specimens. Thus, the examined surface area of fossils came to no less than 3600 m^2^; the specimens were collected from several localities of the Momonoki Formation. Although many plant–insect interactions were found and *Cladophlebis* fern fronds were a dominant component of the fossil floral assemblage, the leaf mines described in this paper were found on only one specimen. Three leaf mines were found on a shale, as one part and counterpart specimen. The fossil type specimen was identified by H.Y. The shale was collected from National Route 435 by H.T. with obtaining permission (Fig. 1). The fossil type specimen is deposited and publicly available in the Mine City Museum of History and Folklore (MMHF), Yamaguchi prefecture, Japan; the catalogue numbers are MMHF11-00001a and MMHF11-00001b. The geological map (Fig. 1) was made by H.Y. based on relevant studies^38,39^, and later slightly modified by Y.I., using Adobe Illustrator® 2021. The field study on fossil plants comply with relevant institutional, national, and international guidelines and legislation.

Photographs of materials were taken by N.O. with a Nikon D850 using three types of Macro-NIKKOR lens (120 mm F 46.3, 55 mm f 2.8, and 65 mm f 4.5). Photos were later edited by Y.I. with Adobe Photoshop® 2021 to increase the contrast and to erase the background. Areas and lengths were measured by Y.I. with Fiji (Fiji Is Just Image J)^110^. For preparing Fig. 4A–F, illustrations were made by Y.I. by tracing photos from the relevant literature with water-based drawing pens, scanned with a CanoScan LiDE 400 (Canon) at a resolution of 300 dpi, and edited with Adobe Photoshop® 2021.

To examine the stoichiometric footprint of this ancient plant–insect interaction, elements of the mine trace were analysed by H.Y. via energy-dispersive X-ray fluorescence (XRF) using a EA6000VX High Sensitivity XRF Analyzer (Hitachi High-Tech Science Corporation). An XRF spectrometer measures elements between Na and U in order of increasing atomic number. Elemental compositions were quantitatively measured from three points of the specimen where organic compounds derived from fossil leaves remained (one point was set on a frass trail of a leaf mine where insect's coprolites were visible; each one sample point was set on leaf vein and lamina of the same pinnule), and, as a control point, from the host sedimentary rock. The measurement duration was set at 120 s using a 0.2 × 0.2 mm collimator under an excitation voltage of 50 kV and a tube current of 1000 μA. Output data were visualized by Y.I. with ggplot2^111^ and formatted with Adobe Illustrator® 2021.

## Supplementary Information

Supplementary Information 1.

Supplementary Information 2.

Supplementary Information 3.

Supplementary Information 4.

Supplementary Information 5.

## Data Availability

The fossil type specimen is housed in the Mine City Museum of History and Folklore (MMHF), Yamaguchi prefecture, Japan: catalogue numbers MMHF11-00001a and MMHF11-00001b. In addition, the original XRF analysis data are provided in Supplementary Information S1–S4.
