# Supplementary material for: Oldest leaf mine trace fossil from East Asia provides insight into ancient nutritional flow in a plant–herbivore interaction
Source: Sci Rep. 2022 Mar 28;12:5254. doi: 10.1038/s41598-022-09262-1 (PMC8960907; doi:10.1038/s41598-022-09262-1)
Supplement: Supplementary file 1 — Supplementary Information 1. [file 41598_2022_9262_MOESM1_ESM.pdf]

Additional Information legends

Supplementary information S1. The raw data of X-ray fluorescence (XRF) analyses on sample point A (frass).

Supplementary information S2. The raw data of X-ray fluorescence (XRF) analyses on sample point B (vein).

Supplementary information S3. The raw data of X-ray fluorescence (XRF) analyses on sample point C (lamina).

Supplementary information S4. The raw data of X-ray fluorescence (XRF) analyses on sample point D (rock).
